# Notch signaling deregulation in multiple myeloma: A rational molecular target

**DOI:** 10.18632/oncotarget.5025

**Published:** 2015-08-13

**Authors:** Michela Colombo, Serena Galletti, Silvia Garavelli, Natalia Platonova, Alessandro Paoli, Andrea Basile, Elisa Taiana, Antonino Neri, Raffaella Chiaramonte

**Affiliations:** ^1^ Department of Health Sciences, Università degli Studi di Milano, 20142 Milano, Italy; ^2^ Department of Clinical Sciences and Community Health, Università degli Studi di Milano; Hematology, Fondazione Cà Granda IRCCS Policlinico, 20122 Milano, Italy

**Keywords:** Notch, multiple, myeloma, molecular, therapy

## Abstract

Despite recent therapeutic advances, multiple myeloma (MM) is still an incurable neoplasia due to intrinsic or acquired resistance to therapy. Myeloma cell localization in the bone marrow milieu allows direct interactions between tumor cells and non-tumor bone marrow cells which promote neoplastic cell growth, survival, bone disease, acquisition of drug resistance and consequent relapse. Twenty percent of MM patients are at high-risk of treatment failure as defined by tumor markers or presentation as plasma cell leukemia. Cumulative evidences indicate a key role of Notch signaling in multiple myeloma onset and progression. Unlike other Notch-related malignancies, where the majority of patients carry gain-of-function mutations in Notch pathway members, in MM cell Notch signaling is aberrantly activated due to an increased expression of Notch receptors and ligands; notably, this also results in the activation of Notch signaling in surrounding stromal cells which contributes to myeloma cell proliferation, survival and migration, as well as to bone disease and intrinsic and acquired pharmacological resistance. Here we review the last findings on the mechanisms and the effects of Notch signaling dysregulation in MM and provide a rationale for a therapeutic strategy aiming at inhibiting Notch signaling, along with a complete overview on the currently available Notch-directed approaches.

## MULTIPLE MYELOMA

Multiple Myeloma (MM) is a hematological malignancy characterized by a malignant proliferation of bone marrow (BM) post-germinal center plasma cells (PCs) and release of monoclonal protein in blood or urine. MM accounts for 1% of all neoplastic disease and represents 13% of hematologic cancers. In Western countries, its annual incidence is approximately 5.6 cases per 100.000 individuals. The median age at diagnosis is about 70 years [1, 2].

**Figure 1:**
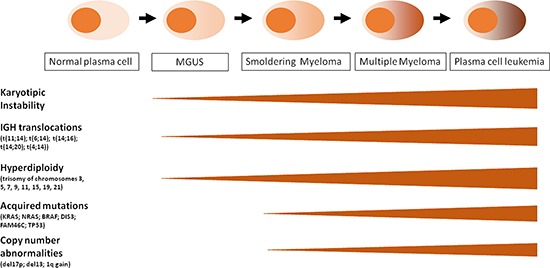
Schematic representation of MM progression and oncogenic events along the four clinical phases: MGUS, SMM, MM, PCL. See details in the text

MM represents a highly biologically and clinically heterogeneous neoplasia; it shows four distinguishable clinical phases that, however, may not be discernible in each patient (Figure 1). MM may be preceded by a monoclonal gammopathy of undetermined significance (MGUS), an indolent, asymptomatic, premalignant phase characterized by a small clonal population (<10%) of PCs within the BM. MGUS may progress to MM at a rate of 1% per year. Intramedullary MM may present as an asymptomatic, smoldering, multiple myeloma (SMM), or associated with organ dysfunction including hypercalcemia, renal failure, anemia, and bone disease. SMM has an average risk of progression to MM of 10% per year [3,4]. The final stage of MM is represented by the plasma cell leukemia (PCL), defined as at least 20% of PCs or an absolute PCs count of more than 2 × 10^9^/L in the peripheral blood. PCL is a rapidly progressive and fatal disease which may occur as secondary (sPCL) in the context of a preexisting refractory MM, or primary (pPCL) if presenting *de novo* in leukemic phase [5,6].

In the last decade, important advances in molecular cytogenetics and global genomic studies of myeloma cells and their normal counterparts have allowed a significant progress in understanding MM pathogenesis, providing the basis for a molecular prognostic classification and the identification of novel potential therapeutic targets. MM is characterized by a profound genomic instability that involves both ploidy and structural rearrangements. Nearly half of MM tumors are defined as hyperdiploid (HD) associated with trisomies of odd chromosomes (including 3, 5, 7, 9, 11, 15, 19, and 21). The remaining tumors are referred as non-hyperdiploid and are frequently associated with the constitutive activation of *CCND1* (11q13), *CCND3* (6p21), *MAF* (16q23), *MAFB* (20q11), or *FGFR3/MMSET* (4p16.3) genes as a result of *IGH* translocations. Generally, HD patients have a better prognosis [7, 8]. Recent data based on whole exome/genome sequencing indicated a heterogeneous pattern of gene mutations in MM, frequently involving member of the ERK pathway (*NRAS, KRAS* or *BRAF)* and, at a lesser extent, other genes such as *DIS3* or *FAM46c* [8–11].

MM is associated with bone disease in more than 80% of MM patients, due to osteoclast-mediated bone destruction which causes hypercalcemia, osteoporosis, bone pain and fractures [12]. In particular, up to 70% of patients have vertebral fractures, which are associated with a high impairment of quality of life, morbidity and mortality [12]. Bone resorption is not only a relevant issue for patients quality of life, but represents also a critical step in the development of this disease, since it supports tumor growth and survival and finally contributes to the development of drug resistance [13, 14].

High incidence of bone lesions in MM patients is due to the ability of malignant PCs to alter the ratio between osteoclasts (OCLs) and osteoblasts (OBLs) in favor of the first [13, 15]. This effect is mediated by an increase of BM-associated anti-osteoblastogenic factors, such as DKK1, IL3, IL7 and TGF-β [11], or pro-osteoclastogenic factors, such as TNFα and RANKL [16, 17, 18]. Importantly, MM cells play a key role in inducing bone disease directly or indirectly, i.e. MM cells may autonomously produce RANKL [16] or stimulate the surrounding BM cells to release RANKL and other soluble factors that promote OCL differentiation [18]. OCLs directly support MM cell proliferation and survival, leading to disease progression [19].

Thus, malignant transformation in MM represents a multistep process due to accumulating genetic and epigenetic alterations of PCs as well as to their aberrant interactions with BM microenvironment.

The use of novel therapeutic agents such as immunomodulators (i.e. thalidomide and lenalidomide) and proteasome inhibitors (bortezomib), as well as the incorporation of high-dose chemotherapy followed by autologous stem cell transplantation represents the current therapy for MM patients up to 65 years old, without comorbidities and organ dysfunction [1, 20, 21]. Conventional chemotherapy (such as melphalan) combined with novel therapeutic drugs is generally administered in patients older than 65 years or unfit [22]. Recently, two different groups of new generation drugs have been developed; these include novel proteasome inhibitors (carfilzomib, ixazomib and marizomib) and drugs with novel mechanisms of action such as monoclonal antibodies, specific inhibitors of signaling pathways and kinases, deacetylase inhibitors and agents activating the unfolded protein response, especially Hsp90 inhibitors [23]. Nowadays, the median overall survival of MM patients is 7–8 years [1].

However, despite the recent remarkable improvements in the treatment of patients and the development of investigational platforms, MM remains still incurable mainly because of intrinsic or acquired drug resistance. MM cells localization in the BM milieu allows the direct interaction with non-tumor BM cells that provide several stimuli promoting neoplastic cell growth and drug resistance, and consequently patient’s relapse [24]. In addition, approximately twenty percent of patients at diagnosis are at high-risk of treatment failure defined by prognostic markers or due to presentation as PCL. The following chromosomal aberrations have been associated with an adverse outcome: i) translocation t(4;14) in 15% patients, which deranges the expression of FGFR3 (a receptor tyrosine kinase) and multiple myeloma SET domain (MMSET; a histone methyltransferase acting as a transcriptional corepressor); ii) translocations t(14;16) and t(14;20) which affects approximately 6% patients resulting in the upregulation of oncogenes c-MAF and MAFB, respectively; iii) the deletion of the short arm of chromosome 17 (del17p) observed in 10% of newly diagnosed MM patients and increasing with MM progression, which results in the loss of *TP53* hampering its role in the control of cell cycle and survival; iv) the gain of the long arm of chromosome 1 which is found in approximately 40% of patients at onset and results in gene amplification of PDZK1, CKS1B and ADAR1 [7–9]. It has been shown that PDZK1 plays a role in conferring drug resistance to MM cells, whereas CSK1B promotes MM cell proliferation and drug resistance through JAK/STAT3 and MEK/ERK [25]. ADAR1 may play a role in tumor progression through its activities of RNA editing, microRNA processing and RNA-induced gene silencing, possibly resulting in malignant reprogramming [26–27].

## NOTCH SIGNALING PATHWAY

A strain of Drosophila characterized by wings with irregular, “notched” margins was discovered in 1919 and lately associated to haploinsufficiency in the Notch gene [28]. In mammals, the Notch family of genes is composed by four transmembrane receptors, characterized by highly homologue sequences (Notch1–4), and two closely related families of membrane-bound ligands: the Delta-like ligands (DLL1, 3, 4) and the Serrate-like ligands (Jagged1 and 2) [29]. The interaction between receptors and ligands induces two proteolytic cleavages, the release of the cytoplasmic portion and the translocation to the nucleus of the active form of Notch (intra-cellular Notch, ICN) [29].

The Notch pathway regulates cell differentiation, apoptosis, proliferation, morphogenesis and it is essential for embryonic development of multicellular organisms [30]. Specifically, in mammals Notch is able to regulate various processes such as vasculogenesis, myogenesis, gliogenesis, neurogenesis and hematopoiesis [29]. Moreover, the Notch pathway is also involved in the homeostasis of adult tissues by regulating cell differentiation [30], promoting stem cells self-renewal [31]and determining cell fate choice in tissue development, including the commitment to T and B cell lineages [32]. The involvement of Notch signaling in the regulation of these important processes may explain the occurrence of the deregulation of Notch receptors or ligands in several types of cancer, including solid (i.e. breast cancer, melanoma, colorectal cancer, glioblastoma, pancreatic cancer) [33] and hematologic tumors (i.e. T-ALL, B-ALL, AML, B-CLL, MM) [34–37].

## THE NOTCH PATHWAY: A KEY MEDIATOR OF MM PROGRESSION

Notch signaling dysregulation in MM can be ascribed to the overexpression of both receptors and ligands (Table 1). In particular, immunohistochemical analyses revealed that Notch1, Notch2 and Jagged1 are highly expressed in primary MM cells compared to low/undetectable levels in non-neoplastic counterparts [38]. Furthermore the increase of Notch1 and Jagged1 expression was reported upon disease progression from MGUS to MM [39]. *NOTCH2* gene expression levels and activity were reported to be increased in the group of MM patients (approximately 6%) carrying the translocations t(14;16)(q32;q23) and t(14;20)(q32;q11) [40]. These translocations result in the activation of two transcription factors (C-MAF and MAFB respectively), responsible of *NOTCH2* transcription [40]. Jagged2 deregulation seems an even more essential step in MM pathogenesis since its overexpression is an early event occurring in the benign MGUS phase [41]. The mechanisms involved in Jagged2 dysregulation are complex and include Jagged2 promoter hypomethylation [41], the aberrant expression of Skeletrophin, an Ubiquitin-ligase necessary for Jagged2 activity [42], and the loss of SMRT/NCoR2 corepressor which results in Jagged2 promoter acetylation and increased transcription [43].

**Table 1:** Alterations of Notch pathway in multiple myeloma

Deregulation type	Phase	Mechanism	Reference
Notch1/Jagged1 expression	Progression from MGUS to MM	unknown	(39)
Notch2 overexpression	MM	Transactivation by MAF genes due to t(14;16) (q32;q23) and t(14;20)(q32;q11)	(40)
Jagged2 overexpression	Since MGUS	Gene expression deregulation due to promoter hypomethylation or loss of SMRT/NCoR2 corepressor. Increased ligand ubiquitination and activity due to aberrant expression of Skeletrophin	(41–43)
HES5 overexpression	MM (LB subgroup)	unknown	(44)
Increased copy number of Notch pathway members	MM (HY subgroup)	Possible mechanism: trisomies of chromosomes 3, 5, 7, 9, 11, 15, 19 and 21	(44)

Finally, with regard to Notch signaling dysregulation in MM, it can be noted that “hyperdiploid” cases are associated with trisomies of different chromosomes [44] at which genes belonging to Notch pathway, such as *NOTCH1* (chr.9q34.3), *NOTCH3* (19p13.2–p13.1), *DLL3* (19q13), *DLL4* (15q14), *MAML1* (5q35), and *MAML2* (11q21), are located. Moreover, high levels of HES5, a direct Notch transcriptional target [45], have been reported in the subgroup of LB patients (low bone disease) [44]. Although these evidences suggest a possible increase of Notch activity in these tumors, they should be further corroborated by a thorough molecular analysis of the expression of Notch signaling members in MM patients subgroups.

**Figure 2:**
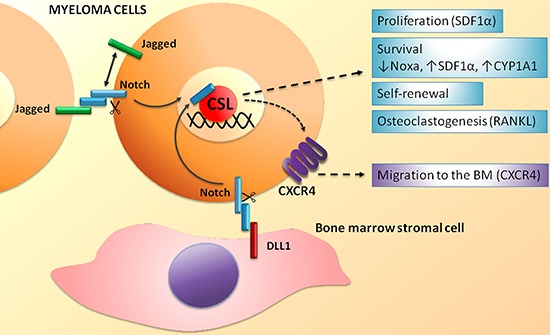
Homotypic and heterotypic activation of the Notch signaling in MM cells Biological effects and molecular effectors activated by Notch signaling in MM cell. See text for details.

The first outcome of Notch receptors and ligands dysregulation in MM is the activation of Notch signaling within tumor cell due to homotypic interaction among nearby myeloma cells or to the engagement of Notch receptors by ligands expressed on the surrounding stromal cells (see scheme in Figure 2). Several reports indicate that Notch inhibition in MM cells induces apoptosis, decreases proliferation rate [46, 47] and increases their sensitivity to pro-apoptotic compounds such as Bcl-2/Bcl-X_L_ inhibitors [48]. Moreover, Notch blockade causes an increase of MM cells sensitivity to standard chemotherapics such as doxorubicin and melphalan both *in vitro* and *in vivo*, finally preventing the development of BM-derived drug resistance [47]. We also showed a role for Notch in the directional migration of MM cell [46] in analogy to other healthy and neoplastic cell types [49–52]. In particular, Notch signaling controls the expression and function of the CXCR4/SDF1α chemokine system, which is crucial in malignant PC growth, survival and migration [46]. Accordingly, *in vivo* inhibition of Notch activity in a xenograft murine model of MM results in the reduction of CXCR4 expression in MM cells and in a consequent significant decrease of MM cell localization to the BM, the primary source of SDF1α chemokine [46]. The evidence that Notch signaling dysregulation may drive MM cell localization at the BM strongly suggests that the increase of Notch signaling activity during MM progression may promote the continuous migration of MM cells from the initially infiltrated BM site to different bone districts, resulting in the formation of multiple bone lesions.

In the last years, another crucial role for Notch signaling has been reported in cancer stem cell self-renewal [53]. The proposed involvement of MM stem cells (MMSCs) in drug resistance, tumor dormancy and relapse has drawn the attention of the scientific community on the identification of specific MMSC markers. Unfortunately, up to now, there are not univocal MMSC markers. Indeed, it is still a matter of debate whether MMSCs originate from B cells, and therefore can be identified as CD138−CD19+CD20+ cells [53], or if they derive from mature plasma cells, which can be found in the CD138+ population [55]. Moreover it cannot be excluded that different MMSC subpopulations rise during MM progression. Even though the lack of unambiguous markers makes the study of MMSCs difficult, a role of Notch signaling in MMSCs has been described on the basis of functional assays [56, 57]. Xu and colleagues reported that MS5 stromal cell line, genetically modified to have a high constitutive expression of human DLL1, increased human and murine MM cells clonogenic growth *in vitro* and accelerated disease development in the 5T33MM murine model [56]. Chiron *et al*. reported that Jagged2 is critical for MM cell self-renewal, showing that spontaneous clonogenic growth of MM cell lines correlated with the expression of Jagged2, whereas on the other end, clonogenic and *in vivo* growth was impaired by Jagged2 silencing [57]. Overall these results suggest that the activation of Notch signaling pathway, mediated by the overexpression of Notch receptors or ligands, may have a key role in promoting MM progression and maintenance.

On the other side, Notch signaling in MM cells can be also activated by Notch ligands expressed on the surface membrane of surrounding BM cells (see scheme in Figure 2). Xu et al. demonstrated that BMSCs expressed DLL1 and were able to engage Notch2 in MM cells. In turn, Notch2 activation caused the upregulation of CYP1A1 (cytochrome P450, family 1, subfamily A, polypeptide 1), contributing to the development of resistance to treatment with bortezomib [58]. Interestingly, the combined treatment with a Notch-blocking agent and bortezomib leads to an increase sensitivity to bortezomib resulting in a significant improvement of the overall survival in an *in vivo* model of MM [58].

**Figure 3:**
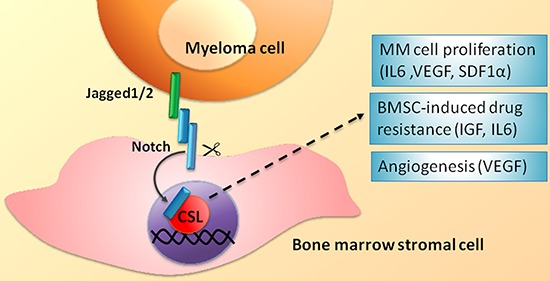
BMSC-mediated heterotypic activation of the Notch signaling in MM cells Biological effects and molecular effectors involved. See text for details.

A second and important outcome of Notch receptors and ligands dysregulation in MM concerns the ability of MM cells to shape the BM niche (see scheme in Figure 3). Jagged2 increases the release of soluble factors including interleukin 6 (IL6), vascular endothelial growth factor (VEGF) and insulin-like growth factor 1 (IGF1) from BMSCs [41]. The released factors have a recognized promoting activity for MM. Indeed, IL6 is the major growth factor for MM cells [59], also involved in the development of resistance to dexamethasone *in vitro* [60]. This is further confirmed by the evidence that Siltuximab, an anti-IL6 monoclonal antibody, gives promising results alone or in combination with dexamethasone in a phase 2 clinical trial on patients with refractory MM [61]. VEGF promotes MM cell growth [62] as well as neo-angiogenesis [63], thereby directly and indirectly promoting tumor burden and progression of MM [64]. IGF1 promotes survival in MM cell and development of bortezomib resistance [65].

**Figure 4:**
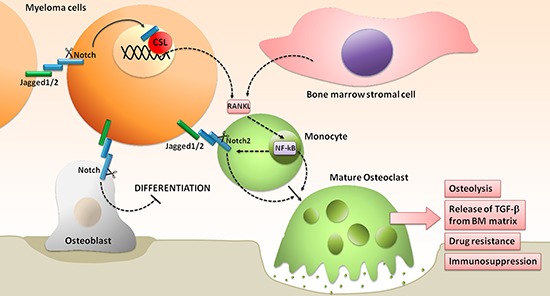
Notch hyperactivation drives the unbalancing of OBLs and OCLs activity, promoting the development of MM-associated bone disease See text for details.

Notch signaling dysregulation in MM plays also a role in MM-associated bone disease, contributing to the unbalance between OCLs and OBLs in favor of the first (see scheme in Figure 4). We recently dissected the different effects due to Notch signaling dysregulation in MM-induced osteoclastogenesis and bone resorption. In particular we showed that: i) high Notch signaling in MM cells stimulates the release of the major osteoclastogenic soluble factor, RANKL; ii) MM cell-derived Notch ligands (Jagged1 and 2) activate Notch signaling in surrounding BMSCs, boosting the secretion of RANKL; iii) RANKL engages RANK on OCL progenitors, thereby activating the osteoclastogenic NF-kB pathway, which in turn stimulates the osteoclastogenic Notch signaling by promoting Notch2 expression; and iv) MM cell-derived Jagged ligands further boost Notch signaling in OCL progenitors by engaging Notch2. Notably, Jagged1/2 silencing is able to revert these effects [66].

Notch hyperactivity in MM may also affect OBL development. Zanotti and co-workers demonstrated in transgenic mice that the selective Notch activation in OBL progenitors inhibits their differentiation [67]. This suggests that MM cell-derived Notch ligands can also be responsible for the decrease of OBLs by stimulating Notch activity that hampers OBL progenitors differentiation.

These results, confirmed *in vivo* by the experiments of Schwarzer and colleagues [68] with γ-secretase inhibitors (GSIs), suggest that the Notch pathway may represent a suitable target for the treatment of MM-associated bone disease. Moreover, the tight cooperation of Notch and NF-kB pathways (mediated by RANKL) in MM-induced osteoclastogenesis suggests that a combination treatment of Notch and proteasome inhibitors could be even more effective. Interestingly, this combined approach could not only affect osteolysis, but also overcome MM-associated drug resistance. Indeed, as reported by Xu and coworkers, GSI-mediated inhibition of Notch2 in MM cell increases the sensitivity to bortezomib [58]. These findings provide a rationale for a combined treatment with Notch and proteasome inhibitors that could synergistically prevent bone disease and drug resistance. Taken together the data reported above suggest that the Notch pathway may represent a rational target for MM therapy, alone or in combination with standard of care chemotherapics.

## NOTCH TARGETED THERAPIES: STATE OF THE ART AND FUTURE PERSPECTIVES

As reported, Notch signaling alterations described in MM result in an increased expression of Notch pathway members including receptors and ligands [42]. These alterations cause a moderate increase of Notch signaling in comparison to Notch pathway hyperactivation occurring in other types of cancer characterized by gain-of-function Notch mutations, i.e. T-ALL [36], other lymphoid malignancies [34] and solid tumors, including breast cancer [69], ovarian cancer [70] and esophageal squamous cell carcinoma [71]. It must also be noted that if a moderate activation of Notch has a pro-tumor effect in MM cells, a stronger activation due to a mutated constitutively active Notch results in MM cell growth inhibition and apoptosis [72]. Recently, Kannan and colleagues [73] provided a possible explanation for this behavior, revealing that the transcription of the Notch target Hairy/Enhancer of Split1 (HES1), occurring upon Notch activation, may have a different outcome depending on the relative levels of HES1 and Poly ADP-Ribose Polymerase1 (PARP1). Indeed, the interaction between HES1 and PARP1 inhibits HES1 growth promoting function and induces PARP1 activation resulting in consequent apoptosis. Consistently, T-ALL cells that carry mutated hyperactive Notch1 and low levels of PARP1 display high levels of cell proliferation and do not undergo apoptosis. Concerning MM cells, we can speculate that, similarly to B-ALL cells, they cannot bear high Notch signaling activation due to the relatively high expression of PARP1 [73].

Although MM cell growth is affected not only by Notch withdrawal but also by its hyperactivation, a therapeutic approach based on Notch inhibition appears to be safer in consideration of the oncogenic activity of Notch in several cell types. A therapeutic approach directed to inhibit Notch signaling in MM relies on previously detailed *in vitro* and *in vivo* evidences of the outcomes of Notch signaling inhibition, including decreased MM cell proliferation [46, 47], stemness [56, 57], migration and BM infiltration [46], MM-associated bone disease [66–68] and an increased sensibility to pharmacological treatments [47, 48, 58].

On the other side, despite the critical role of Notch signaling in MM, the wide genetic heterogeneity might give origin to molecular MM subtypes with different sensibilities to an anti-Notch treatment.

Although a more comprehensive analysis should be addressed to define the putative MM patients who could benefit from a Notch-targeted approach by correlating the molecular characterization and the outcome of Notch withdrawal in primary cells, we can speculate that a suitable subgroup could be MM patients with MAF translocations and consequent high Notch2 levels [40]. A complete gene expression analysis of Notch pathway members could help in evaluating if other patients subgroups (i.e. LB and HY; ref. 44) could be possible candidates for a Notch tailored therapy.

Another aspect that should be considered is the possible resistance of MM cells to anti-Notch treatment. GSI-resistance, caused by mutations occurring in downstream Notch mediators or by epigenetic mechanisms, is well described in T-ALL [74, 75, 76], nonetheless up to now it has not been reported in MM cells.

A Notch-directed therapy in MM can get advantage by the recent development of several compounds targeting the different components of the Notch pathway (Table 2). The most widely used drugs inducing Notch withdrawal are the GSIs. The γ-secretase complex is responsible for the proteolytic cleavage that allows the intracellular portion of the Notch receptor to translocate to the nucleus and activate the transcription by the CSL (CBF1/RBP-J, Su(H), Lag1) nuclear factor [77]. We report the outcome of different GSIs in clinical trials for cancer therapy.

**Table 2:** Clinical trials of Notch pathway inhibitors

Class	Drug(s)	Target	Phase	N. patients	Primary endpoint	Subjects	Trial status	Trial ID (Refs.)
γ-Secretase Inhibitors (GSIs)	RO4929097 +Temsirolimus	γ-Secretase	I	18	Side effects; best dose.	Advanced solid tumors	Completed	NCT01198184 (92)
	RO4929097		II	37	Overall survival; progression free survival.	Metastatic colorectal cancer	Completed	NCT01116687 (90)
	RO4929097 + Cediranib maleate		I	20	Recommended phase II dose;Incidence of adverse events.	Advanced solid tumors	Completed	NCT01131234 (91)
	RO4929097 + Gemcitabine hydrochloride		I	18	Recommended phase II dose.	Advanced solid tumors	Completed	NCT01145456 (93)
	MK0752		I	103	Safety; tolerability; maximum tolerate dose.	metastatic or locally advanced breast cancer; advanced solid tumors.	Completed	NCT00106145 (82)
	MK0752 + Tamoxifen or Letrazole		Pilot study	22	Safety; tolerability.	Early stage hormone receptor-positive breast cancer	Ongoing, not recruiting	NCT00756717(83)
	MK0752 + Docetaxel		I/II	30	Dose limitating toxicity; maximum tolerate dose.	Locally advanced or metastatic breast cancer	Completed	NCT00645333 (84)
	PF-03084014		I	23	First cycle dose limiting toxicities.	Advanced solid tumors	Completed	NCT01286467 (88)
Monoclonal antibody	OMP-21M18	DLL-4	I	30	Safety.	Solid tumors.	Completed	NCT00744562 (106)
	REGN421 (SAR153192)	DLL-4	I	83	Safety; tolerability.	Patients with advanced solid malignancies	Completed	NCT00871559 (107)
	OMP-52M51	Notch1	I	33	Safety; pharmacokinetics; preliminary efficacy.	Relapsed or refractory solid tumors	Recruiting	NCT01778439
	OMP-52M51	Notch1	I	53	Safety; immunogenicity; pharmacokinetics; biomarkers; preliminary efficacy.	Relapsed or refractory lymphoid malignancies	Recruiting	NCT01703572
	OPM-59R5 + nab-Paclitaxel+ Gemcitabine	Notch2Notch3	Ib/II	24	Dose limiting toxicities; progression free survival.	Stage IV pancreatic cancer	Recruiting	NCT01647828 (102)
	OMP-59R5	Notch2Notch3	I	44	Safety; immunogenicity; pharmacokinetics; preliminary efficacy.	Advanced solid tumors	Active, not recruiting	NCT01277146
	OMP-59R5	Notch2Notch3	Ib	80	Dose limiting toxicities; Progression-free survival.	Stage IV Small Cell Lung Cancer	Recruiting	NCT01859741 (103)

Data are from https://clinicaltrials.gov

MK0752 (Merck Chemicals Ltd) is a powerful non-competitive oral GSI and represents the clinical analogues of the compound MRK003. MRK003 has cytotoxic and pro-apoptotic effects on non-Hodgkin lymphoma and MM cells *in vitro* and *ex vivo* and is able to overcome the protective effects of BMSCs [78]; it gave also promising results on *in vivo* xenograft models of uterine serous carcinoma and breast cancer [79, 80]. In the last years, MK0752 has been clinically tested for T-ALL treatment with poor results [81], while a trial on patients with metastatic or locally advanced breast cancer or advanced solid tumors, using intermittent MK0752 dosage, resulted in Notch pathway inhibition associated to good tolerability and clinical benefits (ref. 82; Trial ID NCT00106145).

Recently, MK0752 has been studied in combination with other chemotherapics. A combination with tamoxifen or letrozole gave promising results in the treatment of early stage hormone receptor-positive breast cancer (ref. 83; Trial ID NCT00756717), while a combination with docetaxel has been used for the treatment of patients with locally advanced or metastatic breast cancer and resulted effective in the depletion of the cancer stem cell population (ref. 84; Trial ID NCT00645333).

PF-03084014 (Pfizer Oncology) is a small non-competitive and reversible GSI. This compound has shown a significant antitumor activity in several T-ALL cell lines [85] and in breast cancer xenograft mice [86]. Moreover, PF-03084014 in combination with fludarabine displayed a synergistic effect on cell proliferation and chemotactic response of primary chronic lymphocytic leukemia cells [87]. PF-03084014 is currently tested in phase 1 trial in patients with advanced solid tumors (ref. 88; Trial ID NCT01286467).

RO4929097 (Roche) was tested in phase I study with positive results in single cases of metastatic melanoma, colorectal adenocarcinoma, epithelioid sarcoma out of 92 patients with advanced solid tumors [89]. In phase II clinical trial involving patients with metastatic colorectal cancer, RO4929097 did not change radiographic response and time to progression at the used dose and schedule (ref. 90; Trial ID NCT01116687).

RO4929097 was also tested in combination with different chemotherapics or targeting agents like cediranib (VEGF inhibitor; ref. 91; Trial ID NCT01131234), temsirolimus (a mTOR inhibitor; ref. 92; Trial ID NCT01198184) and gemcitabine (ref. 93; Trial ID NCT01145456). These combination studies, despite the presence of a high percentage (~50–70%) of patients with stable disease, resulted in the reciprocal interference of the used drugs causing the interruption of trials [94].

Independently from their efficacy, the most relevant objection for the use of GSIs is that these drugs do not exclusively target the Notch pathway. Indeed, the GSIs affect γ-secretase which regulates the functions of several substrates such as E-cadherin, N-cadherin [95] and syndecan-3, ErbB4 and CD44 [96]. Moreover, GSIs inhibit the activation of all the four Notch isoforms, thereby affecting all the physiologic functions mediated by the Notch pathway. This may explain the presence of relevant adverse events as fatigue, skin disorders, headache, hypophosphatemia and severe gastrointestinal toxicity [89]. Gut toxicity is the most serious GSI-associated side effect, due to goblet cell metaplasia in the intestinal crypts [97].

Attempts to decrease GSIs toxicity without affecting their efficacy had some successful results using an intermitting dosage schedule in combination with corticosteroids [94]. Additionally, in the last years, new generation drugs were developed for a more specific Notch directed therapy with the purpose of overcoming GSI toxicity. Indeed, recent findings encourage the use of approaches directed to selectively inhibit Notch receptors or ligands. As a matter of fact, whereas it is recognized that the inhibition of both Notch1 and Notch2 induces intestinal goblet cell metaplasia [98] resulting in a major gastrointestinal toxicity, a recent report indicates that this side effect can be avoided through the selective inhibition of Notch signaling activation mediated only by one Notch receptor or one family of Notch ligands (Jagged or DLL). Indeed, antibodies against Notch1 or Notch2 [99], or Notch decoy molecules that selectively disrupt Notch/Jagged or Notch/DLL interaction [100], did not or mildly affect intestine differentiation.

In general, compounds for Notch directed-therapy may be divided into two classes: monoclonal antibodies and molecular drugs.

### Monoclonal antibodies

Monoclonal antibodies (mAbs) were recently developed against both Notch receptors and ligands. mAbs are specific for single members of the Notch family; nonetheless their delivery in solid tumors can be highly problematic, due to their high molecular weight. Thus, anti-Notch antibodies are more frequently used in studies on hematopoietic malignancies [101]. Notch targeting mAbs have entered into early phase clinical development as reported below.

OMP-59R5 (Tarextumab, OncoMed Pharmaceuticals-GlaxoSmithKline) is a fully humanized antibody that targets Notch-2 and 3 receptors [102]. It was tested in combination with nab-paclitaxel and gemcitabine in a still ongoing trial on patients with untreated metastatic pancreatic cancer and other epithelial tumors giving preliminary promising results (Trial ID NCT01647828). Phase I clinical studies are also conducted on advanced solid tumors (Trial ID NCT01277146) and untreated extensive-stage small-cell lung cancer (ref. 103; Trial ID NCT01859741).

OMP-52M51 (Anti-Notch1, OncoMed Pharmaceuticals-GlaxoSmithKline) is a novel antibody specific for Notch-1 that blocks the activation of this receptor by binding its negative regulatory region. It was shown to improve the survival of T-ALL xenograft mice [104] and it is currently under study in two Phase I clinical trials in advanced lymphoid malignancies (NCT01703572) and in solid cancers (NCT01778439). OMP-52M51 and OMP-59R5 have not been tested yet for MM treatment, although they promise to be effective on patients characterized by high levels of Notch1 and Notch2 expression.

OMP-21M18 (Demcizumab, OncoMed Pharmaceuticals), REGN421/SAR153192 (Enoticumab, Regeneron Pharmaceuticals) are humanized antibody-based drugs against the Notch ligand DLL4, which is key in vessels formation [105]. These novel drugs have been developed and successfully tested to contrast tumor-associated angiogenesis giving promising results in phase I clinical trials on advanced solid tumors, causing a reduction in tumor size with a general good tolerability (ref. 106, Trial ID NCT00744562; ref. 107, Trial ID NCT00871559).

The evidence that angiogenesis is increased in the BM of MM patients in correlation with MM cell infiltration, growth and survival [64], provides the rational for a DLL4-based anti-angiogenic therapy in this tumor; nonetheless it must be noted that trials with bevacizumab in MM [108] gave limited results and do not encourage an anti-angiogenic approach in MM.

Recent data in the literature highlight the possibility of affecting other biological features including drug resistance and bone disease by targeting Notch ligands in MM. Several evidences justify a therapeutic approach directed to Notch ligands. Indeed, BMSC-derived DLL1 promotes MM cells growth and resistance to bortezomib [56, 58], Jagged2 overexpression in MM may induce the expression of proliferative and survival factors such as IL6, VEGF and IGF1 [41]; and MM-derived Jagged1 and 2 play a crucial role in MM-induced osteolysis [66]. Currently, the relative antibodies have been generated and now are in preclinical studies [109].

### Molecular drugs

In comparison to antibody-based drugs, molecular drugs are more easily deliverable. SAHM1 is a stapled peptide derived from Mastermind-like (MAML) protein, able to block the canonical Notch signaling and has a therapeutic potential in hematopoietic tumors, indeed it is able to induce apoptosis in MM and T-ALL cells [46, 110].

Recently, Kangsamaksin *et al.* developed new decoy peptides based on Notch1 EGF-like repeats fused to human IgGγ heavy chain (Fc). These decoys may selectively inhibit DLL4 or Jagged1 without affecting the signal mediated by other ligands. The authors showed their efficacy against melanoma, breast, lung and pancreatic cancer both *in vitro* and *in vivo*, including a significantly reduced renal, gastrointestinal and hepatic toxicity, compared to GSI treatment [100].

## CONCLUSIONS

Current studies on Notch pathway in MM indicate that the dysregulated Notch receptors and ligands increase the activation of Notch signaling in MM cells promoting their progression and the ability to shape a supportive surrounding microenvironment. The contribution of aberrantly activated Notch signaling in drug resistance and development of MM associated bone disease provides a strong rational for a therapeutic approach in MM directed to control Notch activity in both malignant plasma cells and the surrounding BM stroma. In our opinion, among all the Notch-targeted therapeutic approaches available or in development, those specifically directed to Jagged1 and 2 promise to be effective and prevent the side effects associated to GSIs.
